# Lane-GAN: A Robust Lane Detection Network for Driver Assistance System in High Speed and Complex Road Conditions

**DOI:** 10.3390/mi13050716

**Published:** 2022-04-30

**Authors:** Yan Liu, Jingwen Wang, Yujie Li, Canlin Li, Weizheng Zhang

**Affiliations:** 1 School of Computer and Communication Engineering, Zhengzhou University of Light Industry, Zhengzhou 450001, China; 2015070@zzuli.edu.cn (J.W.); lcl_zju@aliyun.com (C.L.); zhangwzh@zzuli.edu.cn (W.Z.); 2 School of Artificial Intelligence, Guilin University of Electronic Technology, Guilin 541004, China; yujieli@guet.edu.cn

**Keywords:** autonomous vehicles, lane detection, complex road environment

## Abstract

Lane detection is an important and challenging part of autonomous driver assistance systems and other advanced assistance systems. The presence of road potholes and obstacles, complex road environments (illumination, occlusion, etc.) are ubiquitous, will cause the blur of images, which is captured by the vision perception system in the lane detection task. To improve the lane detection accuracy of blurred images, a network (Lane-GAN) for lane line detection is proposed in the paper, which is robust to blurred images. First, real and complex blur kernels are simulated to construct a blurred image dataset, and the improved GAN network is used to reinforce the lane features of the blurred image, and finally the feature information is further enriched with a recurrent feature transfer aggregator. Extensive experimental results demonstrate that the proposed network can get robust detection results in complex environments, especially for blurred lane lines. Compared with the SOTA detector, the proposed detector achieves a larger gain. The proposed method can enhance the lane detail features of the blurred image, improving the detection accuracy of the blurred lane effectively, in the driver assistance system in high speed and complex road conditions.

## 1. Introduction

With the evolution of advanced assisted driving system and automatic driving technology, the road accident probability is greatly reduced and the driving safety is improved [1,2,3,4]. As a key and challenging part of automatic driving and advanced assistant system, lane detection has also become a research hotspot [5,6,7,8]. It is vital for driver assistance systems to obtain the accurate location of each lane, which is also the goal of the lane detection algorithm. In real scenes, the lane detection model should be able to adapt to a variety of adverse scenarious, such as extreme light (illumination), severe occlusion, atrocious weather and ambiguous lanes [9,10,11]. The lane detection model must overcome various challenges.

In recent years, lane detection algorithms in complex scenarios have made great progress [12,13,14]. Minh et al. [15] proposed an algorithm to improve detection performance mainly by eliminating interference factors (shadows) generated by objects. This algorithm overcomes various illumination problems, especially severe shadows. A neighborhood-based image transformation method was used by Gu et al. [16] to enhance extreme regions, which is robust to light changes and shadow but poorly detected in the presence of other light sources and obstructions. Song et al. [11]. proposed a lane detection method in low light conditions, which uses a convolutional neural network (CNN) [17] and a semantic segmentation network for low light image enhancement and lane line detection, and the combination of the two achieves accurate detection of lane lines in low light environments. Qin et al. [9] designed a simple and effective formula for the speed of the algorithm and challenging scenes, using a large receptive field on the global features, which has good robustness to severely occluded scenes. The current lane detection in complex scenes focuses on lighting, occlusion, while few researchers have addressed lane detection in blurred situations. On the road in real life, potholes on the ground, speed bumps and high-speed driving of cars can easily lead to blurred images. Therefore, lane detection in complex scenarios still faces huge challenges.

Aiming at the difficulty of lane detection in blurred scenarios, a lane detection network for blurred image is proposed in this article. The major contributions in this work are shown as follows.

1. A blurred lane line dataset is provided in this article.
2. An improved GAN is used to enhance the features of the lanes and improve the detection efficiency of blurred lanes in complex road environments.
3. The proposed algorithm performs well in complex road conditions (line curves, dirty lane line, illumination change, occlusions), which is greatly superior to the existing state-of-the-art detectors in high speed and complex road conditions.

## 2. Related Works

At present, a great deal of lane line detection algorithms are used in the autonomous driving field [18,19]. Lane detection algorithms can be divided into two groups: traditional lane detection methods based on basic image processing [20,21,22,23,24,25] and lane detection methods based on deep learning [26,27,28,29,30]. Traditional lane line detection methods often distinguish lane lines from other regions by detecting the basic characteristics of the image, such as linear shapes, color [31,32], and edges. In the traditional lane line detection algorithm, image preprocessing usually includes changing the color space of the image, extracting regions of interest, image equalization, and filtering to eliminate interference. Subsequently, image features are extracted from the preprocessed image, such as color, edge and texture. Edge detection is performed using Canny [33,34] and Sobel operator, and then Hough [35] transformation is used for lane line detection. A lane line detection solution called HistWind is proposed in [36], which does not require powerful hardware support to achieve real-time detection, but it is designed for suburban and highway scenes and is not suitable for other complex scenes. A new lane detection and lane departure warning algorithm is presented in [37], which yields satisfactory results when the lane lines are clearly visible, but encounters difficulties in detection when the lane lines are not visible. Traditional algorithms based on handcrafted features are not always robust in complex environments.

As the rise of deep learning in the field of computer vision, increasing number of neural networks for lane line detection have been proposed [38,39,40]. Lane line detection algorithms based on deep learning can automatically extract the lane line features without making assumptions about the road structure and vehicle state, showing excellent robustness. Many deep learning networks show strong feature extraction and feature processing capabilities in various situations, which have good robustness. Zou et al. [41] proposed a mixed depth architecture employing the integration of CNN and recurrent neural networks (RNN) [42] to investigate lane line detection process in the multiple-frame sequential driving scenes. Since this method has no lane fitting process, the detected lane lines are not very smooth. The dataset used in this paper is constructed based on the TuSimple dataset, and the test follows the TuSimple test criteria with an accuracy of 97.3%. Pan et al. [43] proposed a spatial convolutional neural network (SCNN), extending the traditional layer-by-layer convolution to the layer-by-layer convolution in the feature map, so that information can be transferred more efficiently between pixels. This method of information transfer requires multiple iterations and is likely to cause information loss during long distance propagation. The method uses the CULane dataset and the test result F1 is 0.72. A feature aggregator was proposed by zheng et al. [44] The feature aggregator enriches the lane line features after the ordinary CNN feature extraction. Since the method is based on segmentation, the lane cannot be predicted as a whole, so the smoothness of the lane cannot be guaranteed. The experimental test results of this method on TuSimple and CULane datasets are 96.82% and 0.75, respectively. Aiming at unfavorable driving environments such as rain and night, Seokju Lee et al. [45] proposed an end-to-end multitasking unified network, which is called VPGNet, and it can be used to handle lane line detection and recognition task in real time. However, multitasking learning requires a large amount of additional annotations. The method uses a self-made lane line dataset which includes four scenarios (no rain, rain, heavy rain, and night), and the results show that the F1 scores of the four scenarios are 0.87, 0.79, 0.77, and 0.74, respectively.

Most current lane detection methods are designed to solve the problems caused by illumination and occlusion, while few people address the negative impact of ambiguous situations on lane detection. Hence, an efficient and robust lane detection algorithm for blurred lanes and other complex environments is proposed in the paper.

## 3. Blurred Lane Line Enhancement and Detection Algorithm

**Figure 1:**
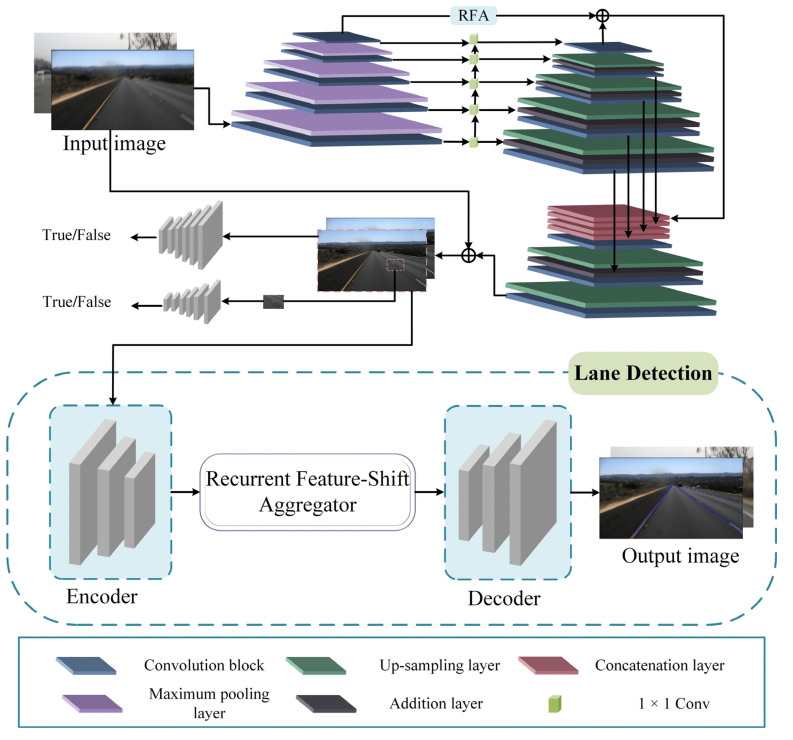
The architecture of proposed Lane-GAN.

This chapter will describe the proposed method in detail, which consists of two parts, a fuzzy image feature enhancement module and a lane line detection module. The overall framework diagram is depicted in Figure 1.

### 3.1. Constructing Blurred Dataset

**Figure 2:**
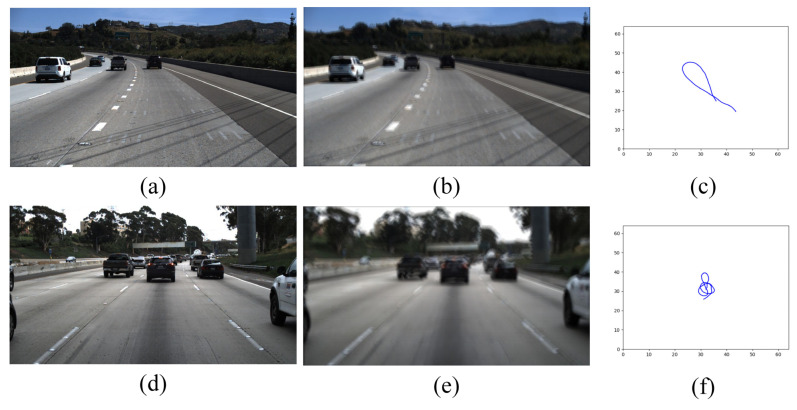
The examples of blurred images in the constructed blur dataset. (**a**,**d**) represent the clear images, (**b**,**e**) represent the blurred images, (**c**,**f**) are the generated camera motion trajectories.

Constructing the fuzzy dataset uses the method of random generation of motion trajectories proposed by Boracchi and Foi [46], where the next position point is randomly generated based on the position, velocity, impulse perturbation, deterministic inertial component, and Gaussian perturbation of the previous point. Then, the trajectory between two random points is generated by sub-pixel interpolation. Each trajectory vector corresponds to a discrete position of a two-dimensional random moving object in a continuous domain. Based on the obtained random trajectory kernel, the blurred image is obtained by applying it to the clear image. The constructed simulated blurred dataset is present in Figure 2.

### 3.2. Blur Image Enhancement

Generative Adversarial Network (GAN) [47] has shown good image enhancement and image restoration capabilities. In this paper, the performance of lane detection algorithm for fuzzy lane lines is improved by enhancing the features of fuzzy lanes using an improved GAN, including a generator and a discriminator.

**Figure 3:**
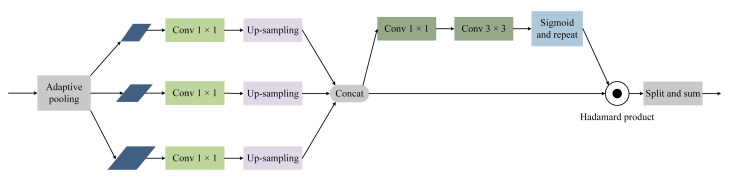
Residual feature augmentation module.

The generator consists of inception-resnet-v2 [48] and an improved feature pyramid network. Feature reuse in the feature pyramid [49] can significantly decrease the calculation time and the size of model. Since the top-level features of the feature pyramid structure are not fused with other features in the top-down stage, the features directly go through a 1 × 1 convolution for dimensionality reduction, the reduction of the number of channels leads to information loss. To compensate the loss of information, the residual feature augmentation module (RFA) is used to perform adaptive pooling on the top layer of the bottom-up phase of the feature pyramid, and then perform 1 × 1 convolutional dimensionality reduction on the feature maps of each scale, followed by up-sampling. Adaptive spatial fusion is performed on the features after up-sampling, and the obtained features are added to the highest layer of the feature pyramid in the top-down stage. The feature enhancement module provides spatial contextual information to reduce the information loss in the feature pyramid, which helps to enrich the features of the blurred image lanes. The residual feature augmentation module is shown in Figure 3.

The discriminator adopts a dual-scale discriminator, which makes full use of global and local features, so that GAN can deal with more complex real lane blurring. Discriminator’s loss function is shown in Equation (1).

(1) $$L_{D}=E_{x∼p_{data}(x)}\left[{\left(D\left(x\right)-E_{n∼p_{n}(n)}D\left(G\left(n\right)\right)-1\right)}^{2}\right]+E_{n∼p_{n}(n)}\left[{\left(D\left(G\left(n\right)\right)-E_{x∼p_{data}(x)}D\left(x\right)+1\right)}^{2}\right]$$

where n represents the noise, G presents the generator, D is the discriminator. $p_{data}\left(x\right)$ represent the probability distribution of the real data x obeys. $p_{n}\left(n\right)$ is the probability distribution of n obeys. $E_{x∼p_{data}(x)}$ and $E_{n∼p_{n}(n)}$ represent expected values.

The generator’s loss function is:

(2) $$L_{G}=0.5\times L_{s}+0.006\times L_{d}+0.01\times L_{ad}$$

$L_{s}$ denotes the mean square error loss, which is to correct the texture and color distortion. $L_{d}$ represents content loss by perceived distance. $L_{ad}$ contains both local and global discriminator loss.

### 3.3. Lane Detection

Lane detection process includes three sections: the encoder, RESA module [44], and the decoder. The encoder uses ResNet [50] for feature extraction. The RESA module moves the sliced feature map in horizontal and vertical directions cyclically, capturing the spatial relationship between rows and columns, so that each pixel can collect global information, which is more beneficial for detecting blurred lanes. The decoder uses a bilateral up-sampling decoder and integrates two branches to recover the low-resolution feature maps into pixel-by-pixel predictions accurately.

**Figure 4:**
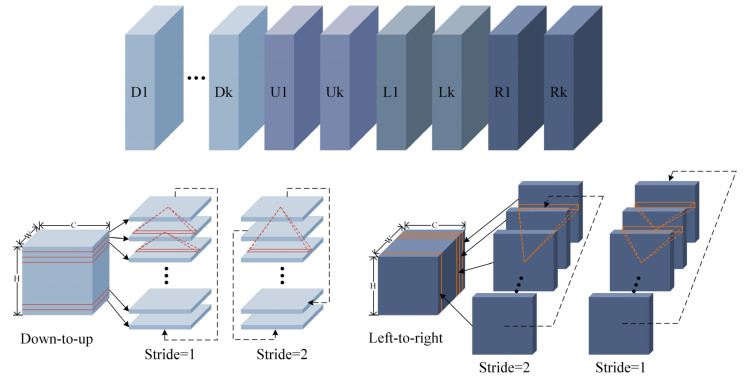
RESA module. D1 to Dk represent k iterations “top to bottom”, U1 to Uk represent k iterations “bottom to top”, L1 to Lk represent k iterations “right to left”, R1 to Rk represent “left to right” for k iterations. In the two modules “bottom-to-top” and “left-to-right”, information is propagated repeatedly and simultaneously at different strides.

RESA module is a recursive feature shift aggregator used to collect spatial features. It first slices the feature map in the vertical and horizontal directions, after which it cyclically moves the sliced feature map in 4 directions (top to down, left to right, down to top, and right to left) so that each slice feature receives another slice feature adjacent to a specific stride. RESA adopts feature shift operation in 4 directions circularly, so that each position can sense and converge all spatial information. Since the serious loss of lane information in the blurred situation, in order to accurately detect the lanes in the blurred situation, we can only rely on the surrounding cues. The RESA module collects features from other places to enrich the feature map, which can well simulate human guessing of the lane, thereby improving the effect of lane detection under ambiguous situations. The RESA module is shown in Figure 4.

Suppose there is a tensor X of three-dimensional feature map of size C×H×W, where H, W and C represent the number of rows, columns and channels. $X_{c,i,j}^{k}$ denotes the feature map X value at the k-order iteration, where c denotes the channel indexes, i and j present the row and column respectively. K is the number of iterations, which is defined as the number of information aggregation to be performed in each direction. Then the forward calculation of recurrent feature shift aggregator is defined as follows:

(3) $$K=\left\lfloor \log_{2}L\right\rfloor$$

(4) $$s_{k}=\frac{L}{2^{K-k}},k=0,1,\cdots ,K-1,$$

(5) $$Z_{c,i,j}^{k}=\sum_{m,n}F_{m,c,n}\cdot X_{m,(i+s_{k})\mathrm{mod}H,j+n-1,}^{k}$$

(6) $$Z_{c,i,j}^{k}=\sum_{m,n}F_{m,c,n}\cdot X_{m,i+n-1,(j+s_{k})\mathrm{mod}W,}^{k}$$

(7) $$X_{c,i,j}^{k\prime }=X_{c,i,j}^{k}+f(Z_{c,i,j}^{k}),$$

where L represents W and H in Equations (5) and (6), respectively. $S_{k}$ represents the move step of the *k*-th iteration. Equations (5) and (6) are the information transfer equations in the vertical and horizontal directions, respectively. F denotes a set of one-dimensional convolution kernels of size $N_{in}$ × $N_{out}$ × W, and W, $N_{in}$ and $N_{out}$ are the kernel width, quantity of input channels and quantity of output channels. The values of $N_{in}$ and $N_{out}$ are the same as C. Z in Equations (5) and (6) is the middle result of information transfer. The feature map X is divided into the horizontal direction with H slices and the vertical direction with W slices. f denotes the nonlinear activation function ReLU. The X is marked by a superscript ′ indicates the updated member.

**Figure 5:**
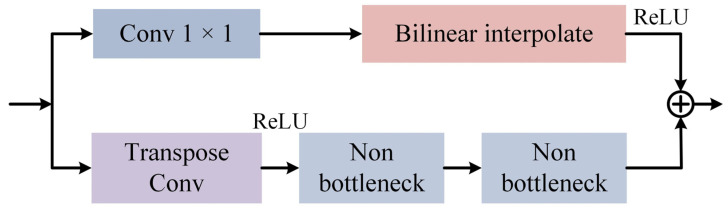
Bilateral Up-Sampling Decoder.

The decoder consists of two branches, one of which is used to obtain rough up-sampling features, and the other for fine-tuning the information loss in the coarse-grained branch. The decoder is shown in Figure 5. The branch to obtain rough features first uses 1 × 1 convolution to decrease the number of channels by half, then batch normalization, followed by bilinear interpolation for up-sampling, and finally ReLU activation function. The other branch is to complement the fine information. The first is to up-sampling the feature map using transposed convolution with a stride of 2 and reduce the number of channels by 2 times, then use the ReLU activation function, and finally stack two non-bottleneck blocks [51].

#### The Loss of Lane Line Detection Module

Lane line detection process is modeled as a semantic segmentation task and a classification problem. The segmentation task is to reduce the difference between the predicted lane segmentation map $S_{pr}$ and the ground truth segmentation map $S_{gt}$. Cross entropy loss is used for the segmentation loss $L_{seg}$ and the segmentation loss is formulated as follows:

(8) $$L_{seg}=L_{CE}\left(S_{pr},S_{gt}\right)$$

where $L_{CE}$ denotes the cross entropy loss. In classification problems, we employ binary cross entropy to monitor the presence of lane lines, predicting the presence or absence of lane lines in an image. The existence loss $L_{exi}$ is expressed by the following equation.

(9) $$L_{exi}=L_{BCE}\left(l_{pr},l_{gt}\right)$$

where $L_{BCE}$ represents the binary cross-entropy loss, $l_{pr}$ denotes the output of the lane presence branch, and $l_{gt}$ is the lane presence label.

## 4. Experiments

### 4.1. Datasets and Evaluation Metrics

**Figure 6:**
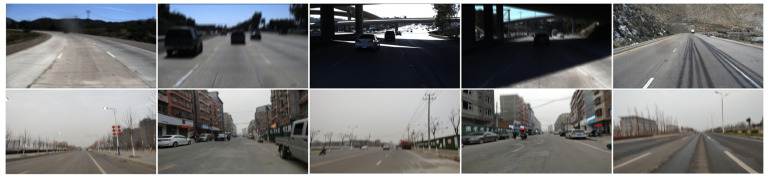
Several examples of blurred images in our dataset.

In order to demonstrate the validity of the algorithm, this paper employs real fuzzy images and simulated fuzzy images. The simulated blurred images are obtained by blurring the images on the TuSimple [52] dataset and the CULane [43] dataset. The TuSimple dataset was captured on US highways with heavily worn lanes and short continuous line segments in relatively good weather and light conditions. The CULane dataset was collected from rural, urban, and highways in Beijing, covering most traffic scenarios and weather conditions. The blurred TuSimple dataset consists of 3268 images in the training set, 358 images in the validation set, and 4173 images in the test set. The test set includes 2782 simulated blurred images and 1391 real captured images. The 1391 real captured images in turn include 835 real blurred images and 556 clear images. The blurred CULane dataset has 88,880 images in the training set, 9675 images in the validation set, and 34,680 images in the test set. This paper will test the algorithm on real captured lane images and simulated fuzzy lane images. Figure 6 displays a brief demonstration of the fuzzy dataset.

This paper adopts the evaluation metrics officially provided by the TuSimple dataset, namely accuracy, false positive rate and false negative rate. The evaluation criterion of accuracy, false positive rate, and false negative rate are calculated as follows.

(10) $$accuracy=\frac{\sum_{clip}C_{clip}}{\sum_{clip}S_{clip}}$$

(11) $$FP=\frac{F_{pre}}{N_{pre}}$$

(12) $$FN=\frac{M_{pre}}{N_{gt}}$$

where $C_{clip}$ denotes the correctly predicted lane points, the mismatch distance between the predicted value and the ground truth value is within a certain scope. $S_{clip}$ represents the amount of total ground truth points in each segment. $F_{pre}$ is the number of lane lines predicted incorrectly, $N_{pre}$ is the total number of lane lines predicted, $M_{pre}$ is the number of lane lines that failed to predict, and $N_{gt}$ is the number of all lane lines in the label.

Traditionally, the F1 metric is used in the CULane dataset. Firstly, the predicted lane line is regarded as the width of 30 pixels, and then calculate the intersection over union (IoU) of the predicted lane line and the lane line marked in the label. Then, according to the set threshold, the lane lines predicted by the network are divided into true positive cases (*TP*), false positive cases (*FP*) and false negative cases (*FN*). The equation of *F*_1_ is calculated as follows.

(13) $$Precision=\frac{TP}{TP+FP}$$

(14) $$Recall=\frac{TP}{TP+FN}$$

(15) $$F_{1}=\frac{2\times Precision\times Recall}{Precision+Recall}$$

The method proposed in this article is based on Python programming and PyTorch deep learning framework. In order to run the algorithm, we built the deep learning environment for PyTorch on the Ubuntu operating system, i.e., CUDA v10.1 + cuDNN v7.6.4 + PyTorch1.4. All experiments are performed on Ubuntu operating system with 32 G RAM and 3.6 GHz CPU, NVIDIA GeForce RTX 2080 Ti 11 GB GPU.

### 4.2. Experimental Results

**Figure 7:**
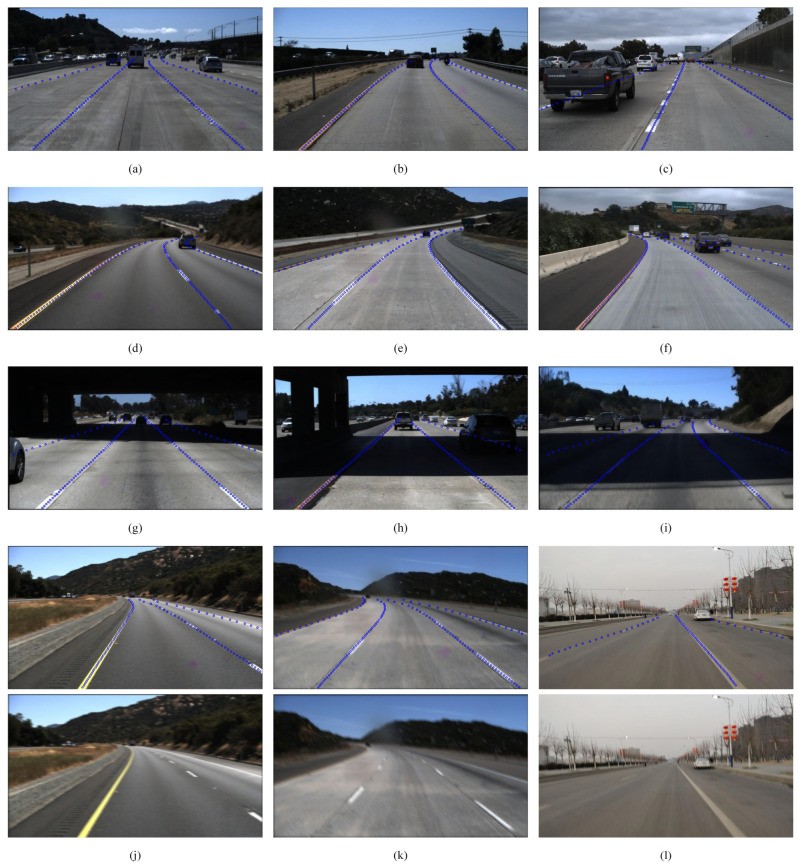
Display the results of Lane-GAN in our dataset. (**a**–**c**) are the detection results when the lane lines are severely worn, the continuous line segment is short, and the lane lines are occluded; (**d**–**f**) present the detection results in the cases of curved lane lines; (**g**–**i**) display the detection results in the cases of shading; the first row in (**j**–**l**) is the detection results of blurred images, and the second row shows the images after enhancement of blurred features.

To illustrate the performance of the proposed network, Lane-GAN was utilized on the test part of constructed dataset. Some test results are shown in Figure 7, which are picked randomly from the test set. Lane-GAN performed well in terms of lane line wear, short continuous line segments, curves, blurring and shadows. It clearly appears that the lane position can still be accurately detected despite the severely worn lane lines, short continuous line segments, and occluded lanes in the Figure 7a–c. The experimental results demonstrate that the detection performance is still good for curved lane lines, which are present in Figure 7d–f. The proposed model is robust when the lane lines are obscured by shadows, as shown in Figure 7g–i. The first row of Figure 7j–l display the detected lanes results of proposed method in blur case, and the second row presents the blurred source images. The experimental results indicate that the method proposed in this paper yields better performance for fuzzy lane detection.

The proposed model not only performs well in the detection of blurred lane lines, but also has good robustness to other complex environments (lane wear, shadows, occlusions).

The comparison results between our algorithm and the state-of-the-art detectors are shown in Table 1. The results demonstrate that the proposed algorithm shows a notable improvement.

As shown in Table 1, the accuracy of CondLaneNet is 92.41%, which is caused by its poor detection of blurred lanes. Although the accuracy of the SCNN method is not too low, it only transmits feature information to adjacent rows or columns, and there is information loss in the process of propagation. The accuracy of UFNet is 92.64%. In UFNet, the constraints of the mask do not exactly match the specified line shapes, so the direct application of conditional instance segmentation to lane detection is not satisfactory. RESA improves the accuracy of detection by repeatedly moving slices of the feature map in the horizontal and vertical directions to aggregate information so that global information is available for each pixel. However, compared with Lane-GAN, RESA has lower detection performance on the constructed dataset, which is due to the ambiguous nature of complex scenes.

To further validate the superiority of the Lane-GAN algorithm, we used the blurred TuSimple dataset to train the comparison algorithms in Table 1 separately, and the test results are shown in Table 2. However, since the CondLaneNet algorithm does not give instructions for training the TuSimple dataset, the CondLaneNet algorithm cannot use blurred TuSimple dataset for training. It can be seen from Table 2 that our algorithm still achieves good results after training with the same dataset.

**Figure 8:**
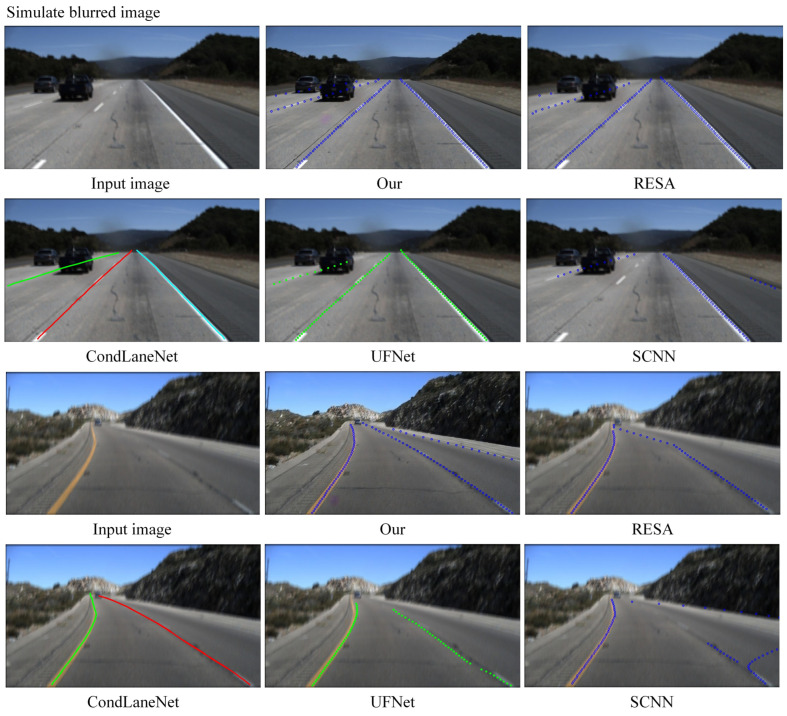

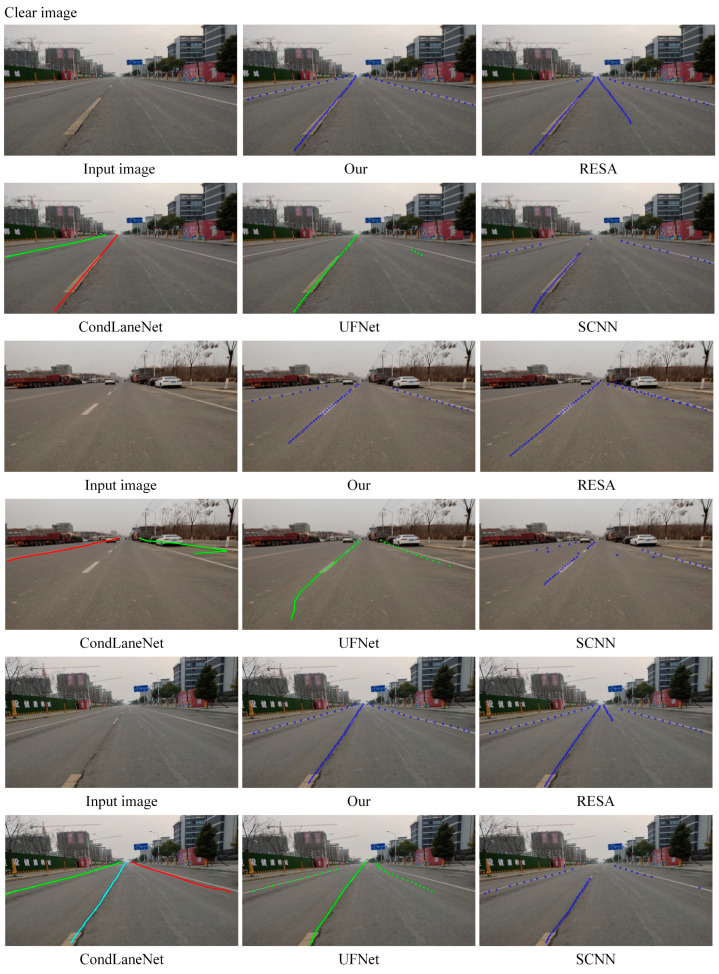

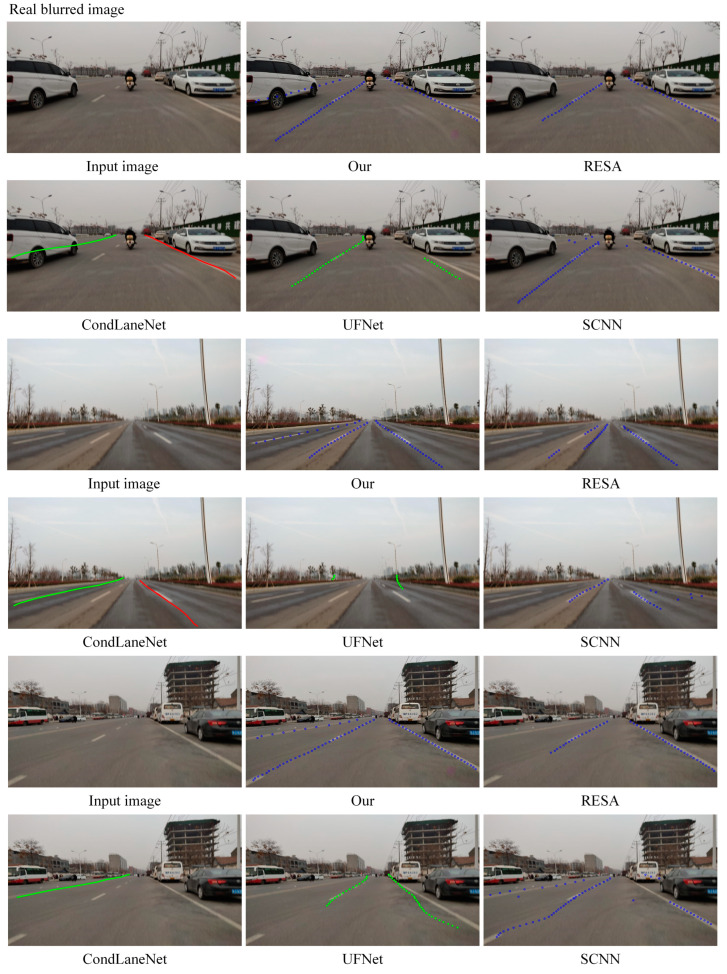
Example of lane detection results based on three sets of images with different detection models.

Figure 8 shows the visualization results of lane prediction with different methods under simulated fuzzy images and real fuzzy images. The proposed network is superior to the existing lane detection methods for the simulated blurred images obviously, especially in heavily obscured and curved areas.

Under the clear images captured in the real scenes, our method can effectively counteract the situation of pseudo lane lines, which are easily detected as lane lines when the road is contaminated mistakenly, while also ensuring smooth detection of all lane lines. For the blurred images captured in the real scene, our algorithm increases the accuracy of lane detection (lanes obscured by vehicles) and decreases the false detection rate of lanes (water stains that are mistakenly detected as lanes). Moreover, it can lessen the disturbance of ambiguous messages and regain detailed information of lanes, which helps to detect lane lines accurately in complex road environments. Lane-GAN achieves the best performance in both sets of images.

The Lane-GAN proposed in this paper has achieved good results but is prone to false detection in the case of rich road markings at intersections, such as zebra crossings. The reason for this is that the zebra crossings have the same appearance as the discontinuous lane lines, and the zebra crossings usually appear at the end of the lane lines. These phenomena can easily cause zebra crossings to be mistakenly detected as lane lines.

In the blurred CULane dataset, the scene complexity of the images can be classified into nine categories. This paper uses the blurred CULane dataset to train the algorithms in the comparative experiments, and the results show that the Lane-GAN algorithm achieves the best detection performance. The results of Lane-GAN and other state-of-the-art methods are shown in Table 3.

**Figure 9:**
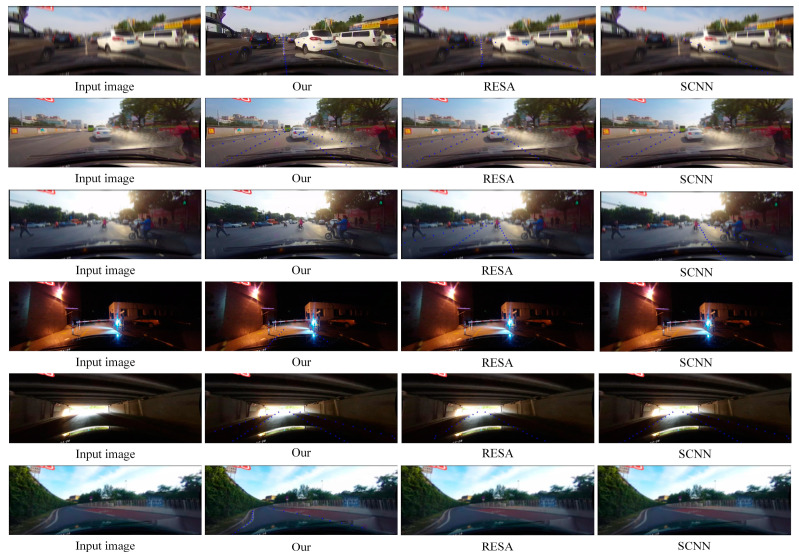
Visualization results of different algorithms on the blurred CULane testset.

Figure 9 shows the visualization results of lane prediction of different methods on the blurred CULane dataset. It can be seen that our method has good detection effect and high robustness in the heavily occluded, dark, curved scenes, and can well resist the complex scenes in the blurred situation, which means that our method has good generalization ability.

### 4.3. Ablation Study

For analyzing the significance of image enhancement for lane detection in blurred scenes, the results of the ablation study are shown in Table 4.

Multiple experimental comparison analysis on the constructed dataset indicated that feature enhancement using blurred lanes improves the accuracy by 0.3% (from 96.26% to 96.56%). It indicates that the Lane-GAN algorithm strongly increases the lane line detection performance of blurred scenes. To address the inevitable problem of blurred lane line detection in real road scenes, a high-precision model is successfully proposed in the paper.

Despite the better results gained by the proposed model, there is still room for improvement in accurately detecting lane positions in complex scenes.

## 5. Conclusions

In the real scene of the road, potholes on the ground, speed bumps and high-speed driving of cars can easily lead to blurred images. Aiming at the problem of unavoidable blurred lane lines in real road scenes, a method Lane-GAN for blurred lane detection is proposed, which addresses the problem of low lane detection rate when lane lines are blurred. First, the blurred image dataset is constructed, then the features of blurred lane lanes in the image are enhanced by using the improved GAN module. Finally, the lane lines are detected. The results of the experiments reveal that the algorithm proposed can increase the detection precision of lane line effectively and lower the false/miss detection rate. The proposed Lane-GAN is robust to water stain, occlusion and blurring. It can also yield excellent results under the real blur condition. In the future, further research on related fields will be studied to keep raising the performance of lane line detection on blurred images.

## Figures and Tables

**Table 1:** The comparison results between the proposed method and the state-of-the-art methods on the constructed dataset.

Methods	Input Size	Accuracy (%)	FP	FN	FPS	Runtime (ms)
SCNN [43]	1280 × 720	91.04	0.1342	0.1600	8	133.5
UFNet [9]	1280 × 720	92.64	0.2625	0.1244	313	3.2
CondLaneNet [53]	1280 × 720	92.41	0.1287	0.1135	220	4.5
RESA [44]	1280 × 720	93.71	0.0682	0.0867	35	28.5
Lane-GAN	1280 × 720	96.56	0.0464	0.0254	7	138.9

**Table 2:** The proposed method is compared with retrained state-of-the-art methods on the constructed dataset.

Methods	Input Size	Accuracy (%)	FP	FN	FPS	Runtime (ms)
SCNN [43]	1280 × 720	95.56	0.0509	0.0547	8	133.5
UFNet [9]	1280 × 720	94.84	0.0445	0.0544	313	3.2
RESA [44]	1280 × 720	96.26	0.0350	0.0368	35	28.5
Lane-GAN	1280 × 720	96.56	0.0464	0.0254	7	138.9

**Table 3:** The comparison results between the proposed method and the state-of-the-art methods on the simulated blurred CULane dataset.

Methods	Input Size	F1	TP	FP	Precision	Recall
SCNN [43]	1640 × 590	70.5	73236	29594	0.7122	0.6982
RESA [44]	1640 × 590	70.1	72020	28712	0.7150	0.6867
Lane-GAN	1640 × 590	72.9	75052	25946	0.7431	0.7156

**Table 4:** Ablation studies of the constructed dataset.

RESA	Improved GAN	Accuracy (%)
√		96.26
√	√	96.56

## Abbreviations

The abbreviations in this paper are as follows:

SOTA	State of The Art
GAN	Generative Adversarial Networks
CNN	Convolutional Neural Network
RNN	Recurrent Neural Network
SCNN	Spatial Convolutional Neural Network
RFA	Residual Feature Augmentation
ResNet	Residual Network
ReLU	Rectified Linear Unit
IoU	Intersection over Union
